# Dietary factors potentially impacting thiaminase I-mediated thiamine deficiency

**DOI:** 10.1038/s41598-023-34063-5

**Published:** 2023-04-28

**Authors:** Katie A. Edwards, Eileen A. Randall, Patricia C. Wolfe, Esther R. Angert, Clifford E. Kraft

**Affiliations:** 1 Department of Pharmaceutical Sciences, Binghamton University, Binghamton, NY 13902 USA; 2 Department of Microbiology, Cornell University, Ithaca, NY 14853 USA; 3 Department of Natural Resources and the Environment, Cornell University, Ithaca, NY 14853 USA

**Keywords:** Bacteria, Nutrition, Ecology, Enzymes

## Abstract

Fish population declines from thiamine (vitamin B1) deficiency have been widespread in ecologically and economically valuable organisms, ranging from the Great Lakes to the Baltic Sea and, most recently, the California coast. Thiamine deficiencies in predatory fishes are often attributed to a diet of prey fishes with high levels of thiamine-degrading (e.g., thiaminase) enzymes, such as alewives, rainbow smelt, and anchovies. Since their discovery, thiaminase I enzymes have been recognized for breaking down thiamine into its pyrimidine and thiazole moieties using various nucleophilic co-substrates to afford cleavage, but these studies have not thoroughly considered other factors that could modify enzyme activity. We found the thiaminase I enzyme from *Clostridium botulinum* efficiently degrades thiamine in the presence of pyridoxine (vitamin B6) as a co-substrate but has relatively limited activity in the presence of nicotinic acid (vitamin B3). Using fluorescence measurements, thiamine degradation in an over-the-counter complete multivitamin formulation was inhibited, and a B-complex formulation required co-substrate supplementation for maximal thiamine depletion. These studies prompted the evaluation of specific constituents contributing to thiaminase I inhibition by both chromatography and fluorescence assays: Cu^2+^ potently and irreversibly inhibited thiamine degradation; ascorbic acid was a strong but reversible inhibitor; Fe^2+^, Mn^2+^ and Fe^3+^ modulated thiamine degradation to a lesser degree. The enhancement by pyridoxine and inhibition by Cu^2+^ extended to thiaminase-mediated degradation from *Burkholderia thailandensis*, *Paenibacillus thiaminolyticus*, and *Paenibacillus apiarius* in tryptic soy broth supernatants. These co-substrate limitations and the common presence of inhibitory dietary factors complement recent studies reporting that the intended function of thiaminase enzymes is to recycle thiamine breakdown products for thiamine synthesis, not thiamine degradation.

## Introduction

**Figure 1:**
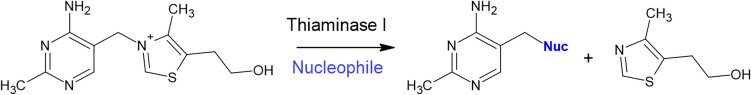
Cleavage of thiamine into pyrimidine and thiazole fragments in the presence of thiaminase I and nucleophilic molecule.

The biological role of thiaminases—enzymes with the capacity to degrade thiamine, an essential vitamin required by all living organisms—has been a mystery since their discovery more than six decades ago^1,2^. Thiamine (vitamin B1) in its diphosphate form is an essential cofactor for enzymes involved in ATP generation via the TCA cycle and pentose phosphate pathway. Thiamine deficiency is causative of beriberi and Wernicke–Korsakoff syndrome, primarily affecting the cardiac and central nervous systems, respectively, in humans^3–11^. Similar disorders have been widespread in aquatic organisms, wildlife, and livestock, yielding the Cayuga, M74, and Early Mortality Syndromes in aquatic organisms; Chastek’s paralysis in foxes; and polioencephalomalacia in sheep and cattle^12–14^. Thiamine deficiencies have been attributed to: (1) an inability to absorb available dietary thiamine (often caused by alcoholism or bariatric surgery)^15^; (2) genetic factors that limit its cellular uptake or processing^7^; (3) restricted intake of thiamine-containing foods (e.g., white rice-based diets)^16^; (4) degradation of thiamine during food processing or storage^17,18^; or (5) the consumption of foods containing chemical constituents or enzymes that break down or complex with thiamine^19^. Enzymatic thiamine degradation was postulated as early as 1941, then later identified as being caused by either of two enzymes, thiaminase I (EC 2.1.5.2) or thiaminase II (EC 3.5.99.2). Thiaminase I enzymes break down thiamine into its pyrimidine and thiazole moieties using various nucleophilic co-substrates, including heterocyclic amines and sulfhydryl groups, to afford cleavage (Fig. 1); thiaminase II uses water as its nucleophile.

Bacteria are known producers of these enzymes, though the contributions of these enzymes to thiamine salvage and synthesis have only been recently clarified^20–23^. The production of thiaminase II in bacteria is more ubiquitous^20,21^; however, only select bacteria are known to produce thiaminase I, including *Paenibacillus thiaminolyticus, Burkholderia thailandensis, Paenibacillus apiarius*, *Clostridium sporogenes*, and *Clostridium botulinum*^23–26^. Thiaminase I has also been reported from various plants and fish^27–30^, although observations from fish have seldom included thorough evaluation of potential microbial origins. Thiaminase activity in the viscera of prey fish has been attributed to the gram-positive bacteria *P. thiaminolyticus*^31–33^, but this bacterium is not consistently found, suggesting the presence of other microbial or endogenous sources^31,34,35^. A recent publication describes the genetic basis of thiaminase activity in zebrafish themselves^35^. The beneficial function of an endogenous thiaminase that would deplete an essential vitamin from its host is not known, nor ﻿are the factors that contribute to enzyme expression and activity or whether this enzyme would contribute to normal or pathological levels of thiamine clearance.

Thiamine deficiency in wildlife populations has been identified as a potential threat to multiple taxonomic groups^36^. For example, managers and scientists have focused on thiamine deficiency as a cause of fishery declines in commercially valuable Baltic Sea^37^, Laurentian Great Lakes^38^, and most recently, U.S. Pacific coast salmon fisheries^39^. These and similar declines have been attributed to thiamine deficiency and consequent mortality in offspring of predatory fish consuming prey with high concentrations of the thiaminase I enzyme^40^.

Thiaminase I-induced thiamine deficiency has been commonly treated for many decades in domestic animals as diverse as sheep and fish through thiamine supplementation. Still, the variable circumstances leading to thiamine deficiency in these organisms have remained perplexing^41^. As thiaminase I requires a nucleophilic co-substrate for thiamine degradation, it is not only the enzyme levels but also the concentrations and identities of endogenous co-substrates that dictate l enzyme activity. Prior studies indicated that adding pyridine, an exogenous co-substrate, to fecal samples of lambs and whole blood samples of crucian carp (*Carassius carassius)* increased their thiaminase activity^14,42^. This indicated that insufficient levels of endogenous co-substrates were present in the samples to support maximal activity of the thiaminase I^42^ and supplementation with saturating concentrations of exogenous substrates allowed the maximum possible thiaminase I activity^14,42,43^. This suggests that thiamine-degrading activity by thiaminase I is influenced by dietary substrate composition and the thiamine degradation that is maximally possible with a co-substrate replete diet. In other studies, prolonged exposure was required to reproduce the symptoms of thiamine deficiency in hatchery fish consuming food with depleted thiamine content from the addition of *P. thiaminolyticus*^44–46^. Similarly, we noted an unexpected resilience of mammalian skeletal muscle cells in culture depleted of thiamine using recombinant thiaminase from *C. botulinum*. These observations prompted our inquiry into the efficiency of thiamine depletion and understanding of thiaminase-mediated thiamine depletion in complex matrices.

In this work, we investigated dietary constituents, including amino acids, vitamins, and minerals, that impact the extent and kinetics of thiamine digestion by thiaminase I from *C. botulinum*. Our studies were carried out with recombinant thiaminase I from *C. botulinum* that was overexpressed in *Escherichia coli* using both pure constituents and over-the-counter vitamin formulations*.* Thiaminase I from *C. botulinum* offers a notable example of this enzyme, with the destruction of thiamine potentially causing an exacerbation of the neurotoxic effects during a botulism infection^47^. *C. botulinum* Type A, B and F strains are known to produce thiaminase I, with Types A and B commonly associated with human disease^24^. Although studies on thiamine and botulism are limited, the available information suggests that thiamine supplementation can reduce toxicity and improve clinical outcomes^48,49^. We also consider the extent to which our primary findings, showing constituents that enhance and inhibit recombinant *C. botulinum* thiaminase activity, apply to thiaminases in culture supernatants produced by *B. thailandensis, P. thiaminolyticus, and P. apiarius*.

## Materials and methods

### Production and characterization of thiaminase I from *Escherichia coli*

The pET-28 plasmid containing the gene for *C. botulinum* thiaminase I (BcmE)^50^ was kindly provided to us by Dr. Chunhao Li and transformed into *E. coli* BL21. This His-tagged thiaminase was overexpressed in *E. coli* and was purified using a Ni-chelate resin using previously reported procedures^51^. The concentration of the purified enzyme was determined using the microBCA assay (ThermoFisher) and purity assessed using SDS-PAGE with Coomassie staining (see Supplementary Fig. S1).

### Screening for thiaminase I natural co-substrates

To determine the effectiveness of various co-substrates towards thiamine degradation, a panel of amino acids and vitamins was incubated with thiamine at 37 °C for 2 h in the presence and absence of thiaminase I. The reactions were then treated with alkaline potassium ferricyanide to oxidize the remaining thiamine to thiochrome prior to fluorescence detection. Thiochrome is the highly fluorescent oxidation product of thiamine, which itself is not fluorescent^52^. Thiaminase-free controls were included to account for constituents that can potentially interfere with the efficiency of thiochrome conversion or yield background fluorescence unrelated to thiaminase. The difference in thiochrome fluorescence intensity from samples treated with and without thiaminase is attributed to thiamine, with the loss of signal corresponding to its degradation. The loss of thiochrome fluorescence was used to determine thiaminase I co-substrate effectiveness. For reactions showing significant activity and dietary relevance, we further monitored thiamine loss due to enzymatic thiamine degradation by LC–MS–MS to confirm observations based on the fluorescence intensity.

Potential co-substrate stock solutions including amino acids and vitamins were prepared at 100 mM in water or DMSO, depending on their solubility at this concentration. These constituents were diluted to 3 mM, thiamine to 30 µM, and thiaminase to 1.5 µg/mL in 20 mM MES/20 mM sodium chloride. These 3× dilutions were combined to yield a solution containing 150 µL of 1 mM co-substrate, 10 µM thiamine, and 0.5 µg/mL thiaminase I in triplicate in a polypropylene microtiter plate. The same compositions with buffer only in lieu of the thiaminase were used as a negative control. 4-Nitrothiophenol (4-NTP), a known efficient thiaminase I co-substrate^48^, was used as a positive control for thiaminase activity, and buffer or DMSO only at the same final dilution as used in co-substrates were included as vehicle controls. We restricted our panel of possible co-substrates to those that exhibited complete solubility at 3 mM in either water or 3% (v/v) DMSO in water. The plate was incubated in a pre-heated incubator at 37 °C for 2 h, unless otherwise specified. 5 µL of the samples were then diluted with 45 µL HPLC grade water in a black, 96-well microtiter plate (Eppendorf), then 100 µL of freshly prepared 0.0075% (w/v) potassium ferricyanide in 15% (w/v) sodium hydroxide (hereafter referred to as alkaline potassium ferricyanide) was added. After rapid mixing, the fluorescence was measured at λ_ex_ = 360/15 nm, λ_em_ = 450/15 nm. GraphPad Prism version 9.4.1 was used for plotting and data analysis.

### Liberation of thiamine from multivitamin supplements

Vitamin B1, Vitamin B Complex, and Multivitamin (Rite-Aid) pills were diluted with 50 mL simulated gastric fluid (0.7% hydrochloric acid, 0.9% (w/v) sodium chloride) and incubated at 37°C with vigorous vortexing every 30 min. over 2 h. Under these conditions, the pills completely disintegrated leaving free floating solids. The 50 mL falcon tubes were centrifuged at 3000×*g* for 5 min. The supernatant was removed and filtered through 0.45 µm pore membranes before further analysis. The composition of these vitamin formulations is listed in Supplementary Table S1.

### Screening for thiaminase I inhibitors

Potential inhibitors were diluted to 3 mM, in a mixture containing thiamine (30 µM) and pyridoxine (3 mM), and thiaminase to 1 µg/mL in 20 mM MES/20 mM sodium chloride. These 3× dilutions were combined in equal volumes to yield a solution containing 1 mM pyridoxine, 10 µM thiamine, and 0 or 0.33 µg/mL thiaminase I in triplicate in a polypropylene microtiter plate. The effect of inorganic ions (Ca^2+^, Co^2+^, Cu^2+^, Fe^2+^, Fe^3+^, Mg^2+^, and Mn^2+^), selected amino acids, and vitamins on thiaminase activity was assessed as above after incubation in a pre-heated incubator at 37 °C for 1 and 2 h, unless otherwise specified. We restricted our panel of possible inhibitors to those that exhibited complete solubility at 3 mM in either water or 3% (v/v) DMSO in water.

To confirm the inhibition from ascorbic acid, 0.33 µg/mL thiaminase and either control buffer or 100 µM ascorbic acid was incubated with varying concentrations of ascorbate oxidase (0.003–0.2 U/mL) for 2 h at 37 °C in 20 mM MES/20 mM sodium chloride. Aliquots of the resulting digests were assayed for thiamine concentration using thiochrome as above.

### Determining the kinetics and concentration-dependence of thiamine degradation and inhibition

The kinetics of thiamine degradation were assessed by diluting pyridoxine and nicotinic acid to final concentrations of 6 nM to 100 µM, in a mixture containing 10 µM thiamine, and 0 or 0.33 µg/mL thiaminase I in triplicate in a polypropylene microtiter plate. Thiaminase activity was assessed by conversion to thiochrome as above after incubation in a pre-heated incubator at 37 °C for 5 min to 4 h. Determination of the *V*_*max*_ and *K*_*m*_ was carried out using GraphPad and a Michaelis–Menten fit.

The kinetics of inhibition were assessed by diluting CuCl_2_ and ascorbic acid to final concentrations of 6 nM–100 µM, in a mixture containing 100 µM pyridoxine, 10 µM thiamine, and 0 or 0.33 µg/mL thiaminase I in triplicate in a polypropylene microtiter plate. Thiaminase activity was assessed by conversion to thiochrome as above after incubation in a pre-heated incubator at 37°C for 15 min. to 5 h. Determination of the IC_50_ was carried out using GraphPad and a 4-parameter inhibitor vs response non-linear fit.

### Chromatographic reaction monitoring

Reactions containing 10 µM thiamine, 100 µM pyridoxine or nicotinic acid and the presence or absence of thiaminase (0.33 µg/mL) in 20 mM MES, 20 mM NaCl, pH 6.5 were incubated for 2 h at 37 °C. To assess reaction inhibition, 100 µM Cu^2+^ or ascorbic acid was also included. The solutions were then transferred to 10 kDa MWCO centrifugal filters (Pall) and centrifuged for 5 min at 10,000×*g* to remove thiaminase. The filtrates were assayed with modifications of the method reported by Verstraete et al.^53^. The method used a Phenomenex Gemini NX-C18 column (100 mm × 3 mm, 3 µm particle size), Waters Xevo TQD LC–MS–MS system, using mobile phases 10 mM ammonium bicarbonate pH 8.8 and methanol. A flow rate of 400 µL/min was used throughout the 8-min run, starting with a linear increase in methanol from 0 to 50% in three minutes, followed by a rapid increase in methanol to 95% over the next 0.5 min. This was maintained for 2 min before rapidly returning to 100% ammonium bicarbonate for 2.5 min for equilibration. UV detection was set at 220 nm and 245 nm. At the end of each day, the column was washed with 100% methanol.

### Determining the impact of inhibitors on other thiaminases

*Burkholderia thailandensis* E264 (ATCC 700388)*, Paenibacillus thiaminolyticus* (B-4156)*, and Paenibacillus apiarius* (B-23460) were cultured in tryptic soy broth (TSB) in a shaking waterbath at 30 °C for 48 h. The *Paenibacillus* spp. were obtained from the USDA NRRL Culture Collection and *B. thailandensis* from the American Type Culture Collection. The OD was monitored at 600 nm. The cultures were transferred to 1.5 mL centrifuge tubes, centrifuged for 5 min. at 16×*g*, and supernatant collected. The supernatant was pooled and filtered through 0.2 µM PVDF syringe filters. The bacterial supernatants were aliquoted and stored at − 80 °C. The high background from sterile TSB did not permit determination of the protein concentration using the MicroBCA assay, so normalization in downstream thiaminase activity assays was on the basis of the OD_600_ taken prior to supernatant collection. The activity of the supernatants at various dilutions made in sterile TSB was assessed using fluorescence intensity measurements made in the presence of a thiamine, cosubstrate and/or an inhibitor. The final composition of 10 µM thiamine, 100 µM pyridoxine or 100 µM nicotinic acid and 0–100 µM Cu^2+^ or ascorbic acid in diluted in 20 mM MES/20 mM sodium chloride, pH 6.5 (two-thirds of the overall reaction mixture) and cultured media diluted with sterile TSB (one-third of the overall reaction mixture) was incubated at 37 °C for one to three hours. 5 µL of the reaction mixture was diluted with 45 µL water, then 100 µL potassium ferricyanide in 15% (w/v) sodium hydroxide was added prior to measurement using 360/40 nm excitation, 450/50 nm emission.

## Results

### Screening for efficient *Clostridium botulinum* thiaminase I co-substrates

**Figure 2:**
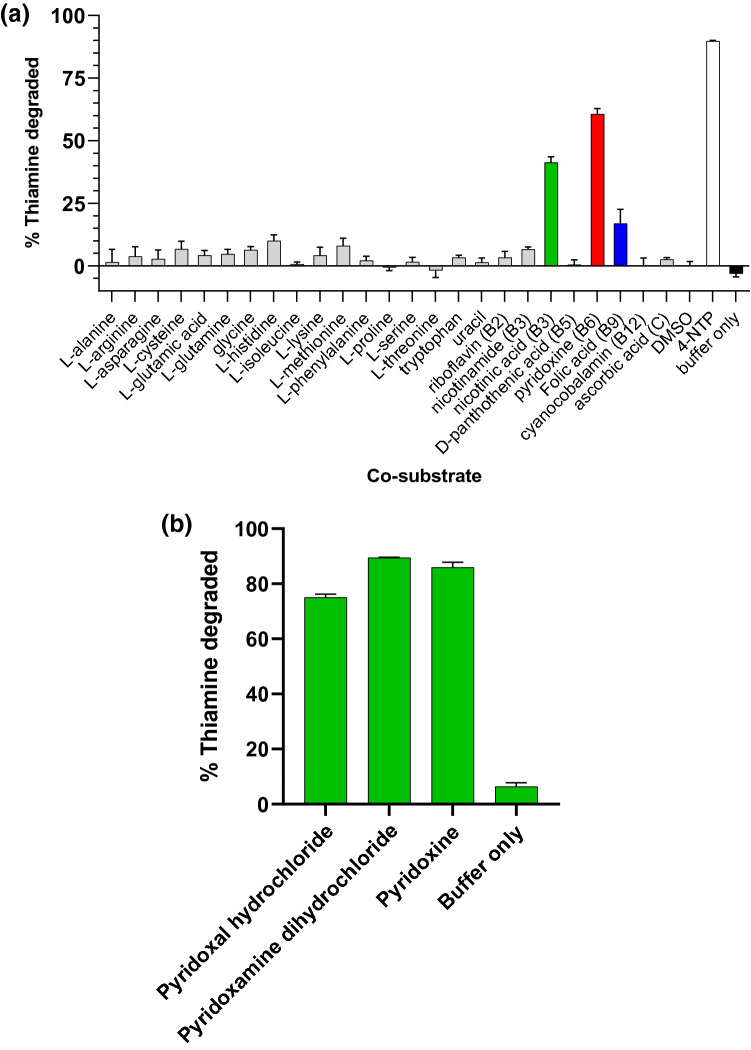
Screening for natural co-substrates for thiaminase I from *C. botulinum* (**a**) amino acids and vitamins and (**b**) congeners of vitamin B6*.* 10 µM thiamine, 1 mM co-substrate in 20 mM MES/20 mM NaCl, pH 6.5 were incubated in the presence and absence of 0.5 µg/mL thiaminase I at 37 °C for 1 h. 5 µL of the reaction mixture was diluted with 45 µL water, then 100 µL alkaline potassium ferricyanide was added prior to measurement using 360/40 nm excitation, 450/50 nm emission. 4-NTP was used as a positive control as a known effective co-substrate.

Naturally occurring co-substrates that yielded degradation in the presence of *C. botulinum* thiaminase I were pyridoxine (vitamin B6), nicotinic acid (niacin, vitamin B3) and folic acid (vitamin B9) (see Fig. 2a, Supplementary Fig. S2). Pyridoxine yielded 60.6% degradation, nicotinic acid yielded 42.5% degradation, and folic acid yielded 16.9% under the same conditions. A small amount of degradation of thiamine was observed in the presence of l-methionine (8.0%) and l-histidine (10.1%). None of the other l-amino acids or vitamins yielded significant degradation of thiamine in the presence of thiaminase, suggesting the presence of dietary pyridoxine or nicotinic acid was required for thiaminase activity.

In a subsequent experiment, the congeners of vitamin B6 (pyridoxine, pyridoxal, and pyridoxamine) were compared, yielding 89.5% degradation in the presence of pyridoxamine, 86% degradation with pyridoxine, and 75% degradation with pyridoxal (Fig. 2b). Nicotinamide, which differs from nicotinic acid by an amide group in lieu of a carboxylic acid group, was ineffective as a co-substrate. This indicated that small differences in co-substrate structure have a marked impact on thiaminase I activity.

The positive control, 4-nitrothiophenol (4-NTP), a known effective co-substrate for many thiaminases^27,54^, was the most effective co-substrate in our experiments. However, it should be noted that this compound, in the absence of thiaminase, suppressed either thiochrome formation or thiochrome fluorescence relative to buffer only (− 33.4%) (see Supplementary Fig. S2). This highlighted the necessity to measure thiochrome values in the absence of thiaminase to serve as a control to compensate for variable thiochrome responses in the matrix. The co-substrate efficacy results were interpreted as a percent change of fluorescence (Fig. 2b) in the presence versus absence of thiaminase for the respective co-substrate (see Supplementary Fig. S2).

**Figure 3:**
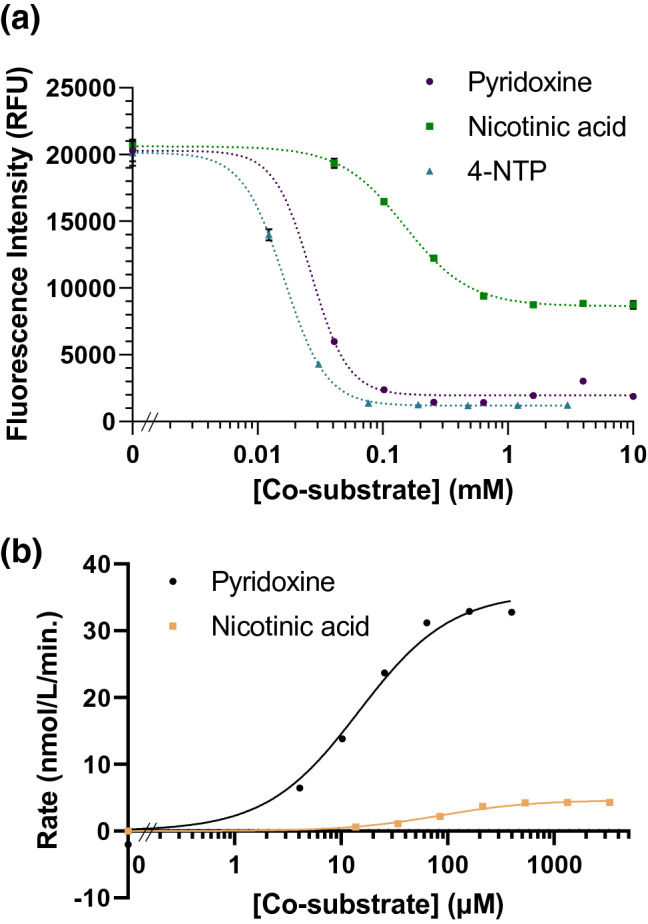
(**a**) Co-substrates for thiaminase I from *C. botulinum.* 10 µM thiamine, 10 nM–10 mM 4-NTP, nicotinic acid, and pyridoxine in 20 mM MES/20 mM NaCl, pH 6.5 were incubated in the presence of 0.5 µg/mL thiaminase I at 37 °C for 24 h. 5 µL of the reaction mixture was diluted with 45 µL water, then 100 µL 0.05% potassium ferricyanide in 15% (w/v) sodium hydroxide was added prior to measurement using 360/40 nm excitation, 450/50 nm emission. (**b**) Rates of thiamine loss after 30 min. as a function of pyridoxine and nicotinic acid concentrations.

We then investigated the co-substrate concentration dependence of thiamine breakdown. Thiamine at a concentration of 10 µM was incubated with thiaminase I and pyridoxine, nicotinic acid, or 4-NTP from 10 µM to 10 mM for up to 24 h at 37 °C. Both 4-NTP and pyridoxine were effective co-substrates, yielding complete thiamine degradation by 100 µM. However, nicotinic acid appeared to be only able to degrade approximately 58% of the available thiamine despite high concentrations (up to 10 mM) of co-substrate and extended time for enzymatic action (Fig. 3a). The incomplete degradation of thiamine with nicotinic acid suggested inhibition of thiaminase I induced by high concentrations of nicotinic acid itself or by the product of the nicotinic acid-thiamine reaction. For pyridoxine and nicotinic acid, *K*_*m*_ values of 14.6 µM and 84.2 µM, and *V*_*max*_ values of 35.8 and 4.6 nmol/L/min, respectively, were observed (Fig. 3b).

**Figure 4:**
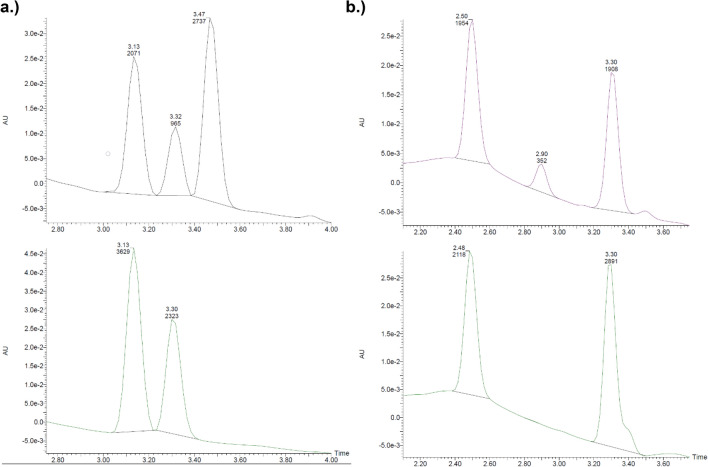
Chromatographic separation of thiamine (retention time = 3.3 min) (10 µM), (**a**) pyridoxine (retention time = 3.1 min, 100 µM) or (**b**) nicotinic acid (retention time = 2.5 min, 100 µM), with (bottom) 0 or (top) 0.33 µg/mL thiaminase for 2 h. The formation of the respective products of pyridoxine and nicotinic acid in the presence of thiaminase is observed at retention times of 3.5 min and 2.9 min, respectively.

As these reactions were monitored by fluorescence alone, overlapping fluorescence spectra of thiochrome with the formed product between the co-substrate and the thiamine pyrimidine ring could not be excluded. We thus monitored these reactions also by chromatographic separation with UV detection (see Fig. 4, Supplementary Table S2). The inefficiency of nicotinic acid (ret. time = 2.50 min) as a co-substrate was confirmed, with only a 34.0% reduction of thiamine peak area (ret. time = 3.32 min) after 2 h at 37 °C, relative to 58.4% with pyridoxine (ret. time = 3.13 min) under otherwise identical conditions. In the presence of thiaminase and pyridoxine, we observed the formation of a new peak at 3.47 min, corresponding to the adduct between the thiamine pyrimidine ring and pyridoxine, and a reduction in pyridoxine peak area by 42.9%. In the presence of thiaminase and nicotinic acid, we observed the formation of a new peak at 2.90 min, corresponding to the adduct between the thiamine pyrimidine ring and nicotinic acid, and a reduction in nicotinic acid peak area by 7.7%. This experiment confirmed that the pyridoxine-thiamine pyrimidine conjugation product is more efficiently formed and that both thiamine and its co-substrate are depleted during thiaminase I-mediated action.

### Activity of thiaminase I in dietary supplement formulations

**Figure 5:**
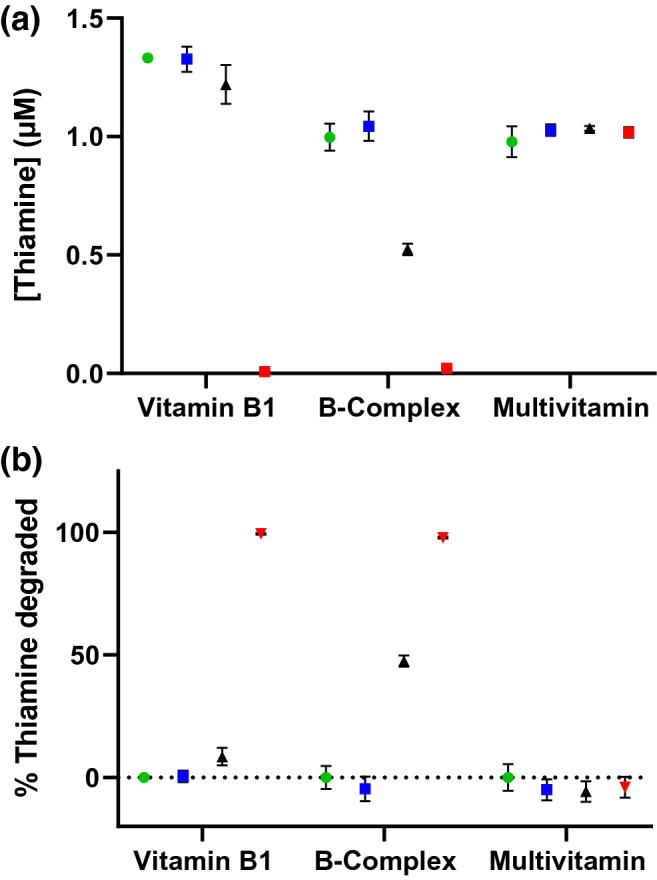
(**a**) Commercial vitamin B1, B-complex, and multivitamin simulated gastric fluid extracts diluted to theoretical 1 µM thiamine and incubated with (green) 0 µg/mL thiaminase and 0 µM pyridoxine, (blue) 0 µg/mL thiaminase and 100 µM pyridoxine, (black) 0.33 µg/mL thiaminase and 0 µM pyridoxine presence and (red) 0.33 µg/mL thiaminase I and 100 µM pyridoxine at 37 °C for 2 h prior to oxidation with alkaline potassium ferricyanide and fluorescence measurement using 360/40 nm excitation, 450/50 nm emission. Each point and error bar represents the average and standard deviation of triplicate fluorescence measurements. (**b**) Data represented as percent thiamine degradation.

To understand these results in a dietary context, we treated commercially available vitamin B1, vitamin B complex, and multivitamin tablets with simulated gastric fluid to liberate the constituents from the formulations. After neutralization and normalization to an equivalent thiamine concentration (1 or 10 µM) based on the label claim, these extracts were incubated with buffer or thiaminase I in the presence or absence of added pyridoxine (100 µM or 1 mM, Fig. 5). The B complex formulation contained six other B vitamins, para-aminobenzoic acid, inositol, choline bitartrate, and cellulose. The multivitamin complex formulation contained numerous other vitamins, minerals, and inactive excipients. The B-complex and multivitamin formulations contained molar ratios of 1.6:1.0 and 2.1:1.0 pyridoxine to thiamine, assuming complete liberation and dissolution. Both formulations contained nicotinamide, rather than nicotinic acid, hence this was not factored in as a co-substrate. The B1 formulation contained only thiamine as an active ingredient along with inactive constituents. The complete compositions of these formulations are provided in Supplementary Table S1.

In all formulations, there was no apparent degradation of released thiamine in the absence of thiaminase I, irrespective of the presence of pyridoxine (Fig. 5). However, in the presence of thiaminase I, the thiamine degradation in the formulations varied significantly. In the vitamin B1 formulation, there was 8.5% degradation of thiamine in the absence of 100 µM pyridoxine and 99.4% degradation in its presence, indicating the necessity of a co-substrate for enzyme activity. The low levels of degradation in the absence of a secondary nucleophile suggest the potential for trace levels of natural co-substrates available in the vegetable source excipients or that thiamine itself can act inefficiently as a co-substrate^55^. In the B-complex formulation, there was 47.5% degradation of thiamine in the absence of 100 µM pyridoxine and 97.9% degradation in its presence. By contrast, no degradation of thiamine was observed in the multivitamin formulation either in the absence or presence of additional 100 µM pyridoxine. In both the B-complex and multivitamin formulations, the excess of existing pyridoxine would yield sufficient co-substrate availability for significant thiamine digestion upon adding thiaminase, and additional pyridoxine would seem unnecessary. However, despite the excess of pyridoxine, thiamine degradation was incomplete or negligible, suggesting that thiaminase I inhibitors were present. These results were confirmed by the fluorescence spectra, showing retention of thiamine in the multivitamin formulation, but degradation of thiamine as a function of pyridoxine concentration in the vitamin B1 and B-complex formulations (see Supplementary Fig. S3).

### Impact of non-cofactor natural constituents on *Clostridium botulinum* thiaminase I activity

**Figure 6:**
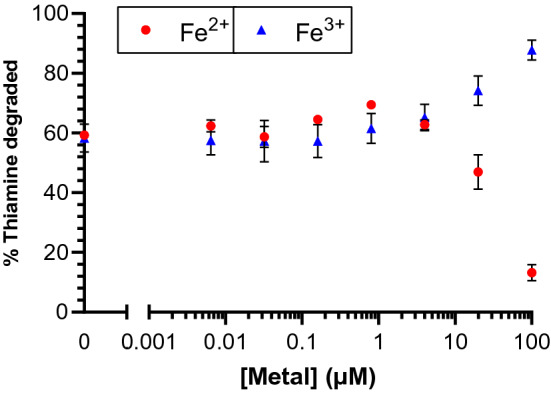
Effect of inorganic ions on thiamine-depletion by thiaminase I from *C. botulinum.* 10 µM thiamine, 1 mM pyridoxine and 0.01–100 µM FeSO_4_ (circles) and FeCl_3_ (triangles) in 20 mM MES/20 mM NaCl, pH 6.5 were incubated in the presence and absence of 0.33 µg/mL thiaminase I at 37 °C for 1 h. 5 µL of the reaction mixture was diluted with 45 µL water, then 100 µL alkaline potassium ferricyanide was added prior to measurement using 360/40 nm excitation, 450/50 nm emission. The results are reported as the percent of thiamine degraded.

To account for the presence of inorganic ions in the multivitamin formulation, and likely in food sample matrices, we investigated the impact of FeSO_4_, FeCl_3_, MgCl_2_, CaCl_2_, MnCl_2_, CoCl_2_, and CuCl_2_ on *C. botulinum* thiaminase I activity. Thiamine was incubated with thiaminase I and pyridoxine in the presence of these inorganic compounds at concentrations from 100 nM to 10 µM for 1 to 21 h at 37 °C. These inorganic compounds themselves had no significant impact on thiochrome formation (see Supplementary Fig. S4) and thus did not pose a detection interference, but they did impact the kinetics of thiamine degradation. The amount of thiamine degraded after one hour was up to 16.7% and 23.5% greater in the presence of Fe^3+^ and Mn^2+^ ions, respectively, suggesting an enhancement in thiaminase activity. By contrast, the amount degraded was up to 13.7% less in the presence of Fe^2+^ ions, suggesting an inhibition of thiaminase activity (see Supplementary Fig. S4). These effects occurred in a concentration-dependent manner, with 100 µM Fe^2+^ markedly reducing thiamine degradation to 13% and 100 µM Fe^3+^ increasing thiamine degradation to 88% versus 60% in the absence of either cation after 1 h (Fig. 6). There was no notable impact of Ca^2+^, Mg^2+^, or Co^2+^ (see Supplementary Fig. S4) ions on enzyme activity under the conditions used, suggesting that these differences were a function of the identity of the cationic species rather than the chloride counter ions. After an incubation period of 21 h, thiamine degradation was complete with all inorganic additives, except Cu^2+^, suggesting that micromolar concentrations of Fe^2+^ slowed degradation kinetics but did not irreversibly inhibit the enzyme.

**Figure 7:**
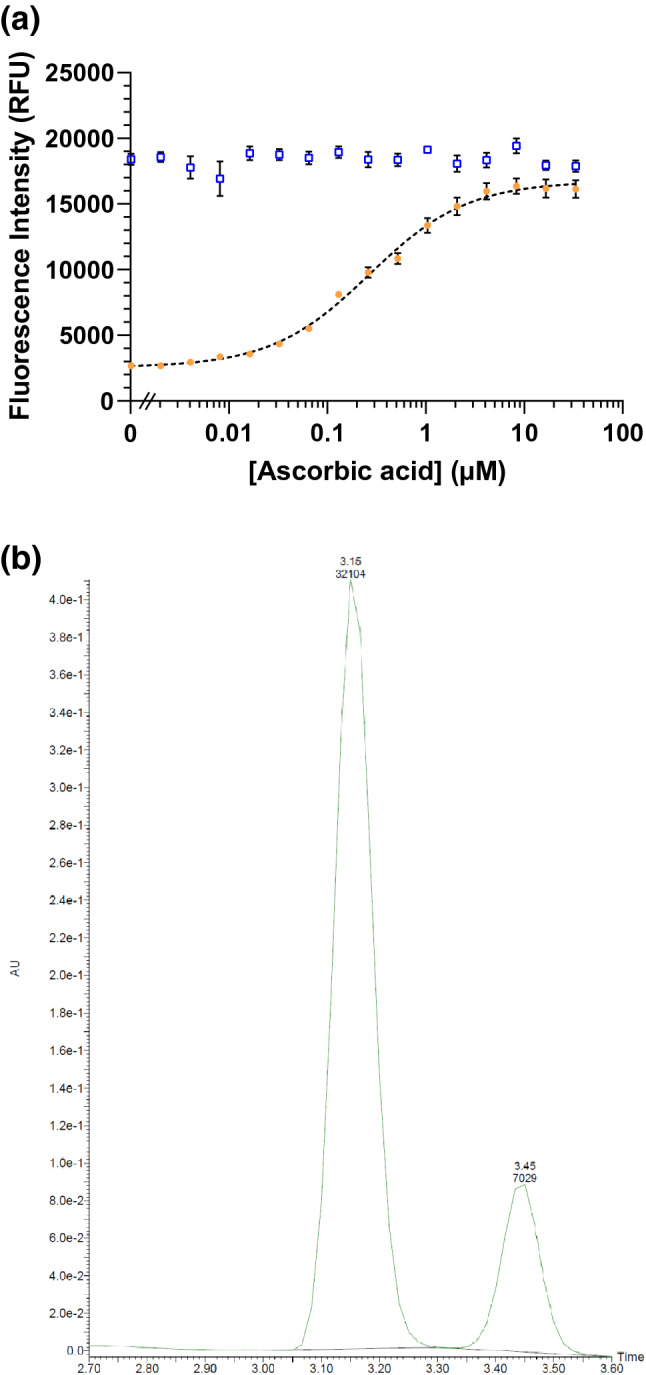

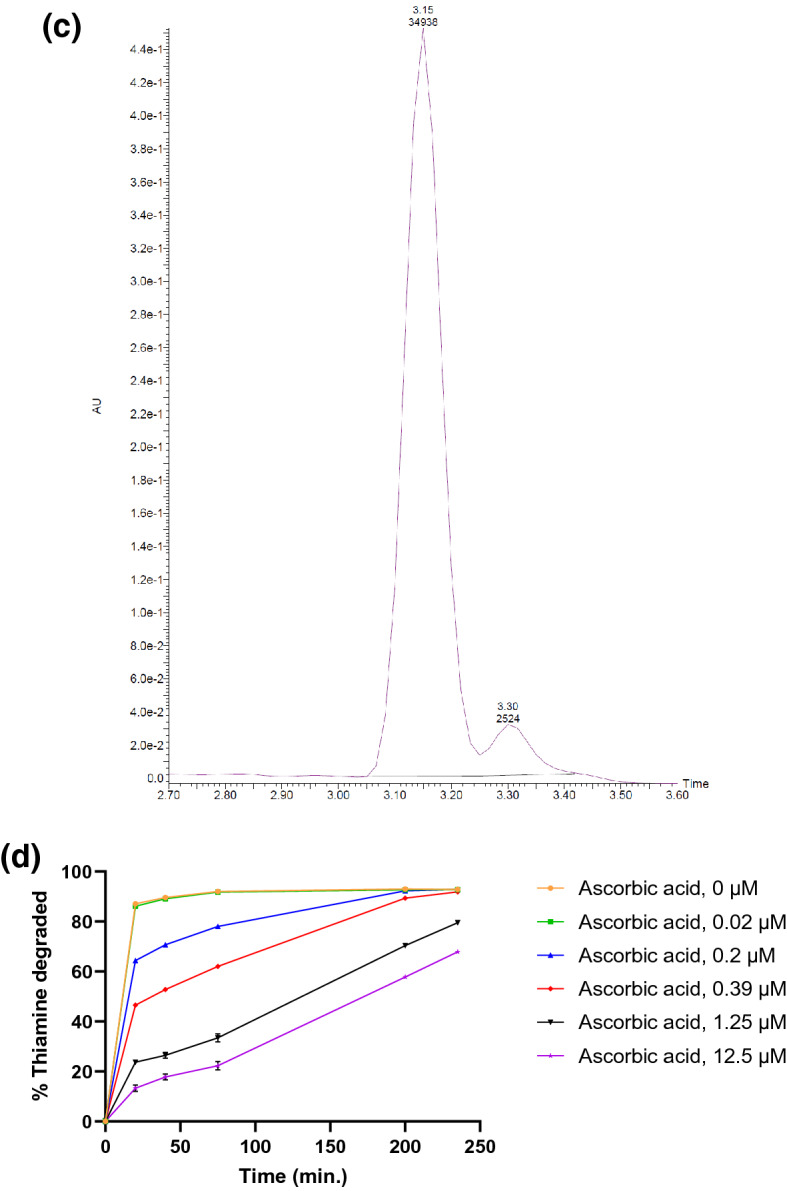
Inhibition of thiaminase I-mediated thiamine degradation in the presence of ascorbic acid. (**a**) Fluorescence intensity measurements using 10 µM thiamine, 1 mM pyridoxine, and ascorbic acid (0–100 µM) incubated in the presence (orange circles) and absence (blue squares) of 0.33 µg/mL thiaminase I at 37 °C for 1 h. 5 µL of the reaction mixture was diluted with 45 µL water, then 100 µL alkaline potassium ferricyanide was added prior to measurement using 360/40 nm excitation, 450/50 nm emission. Each point is the average of the fluorescence values in triplicate with standard deviation represented by the error bars. A 4-parameter logistic equation was used to fit the data (dotted line) from the thiaminase incubations. (**b**) chromatograms showing the formation of the pyridoxine-pyrimidine adduct at 3.45 min and loss of thiamine (3.30 min) under conditions listed above in (**a**) in the absence and (**c**) presence of 100 µM ascorbic acid. (**d**) The kinetics of thiamine degradation obtained using endpoint fluorescence intensity measurements at ascorbic acid concentrations ranging from 0 to 12.5 µM from 15 min to 4 h.

We also screened for inhibition or enhancement of thiaminase activity in the presence of pyridoxine and various natural constituents (see Supplementary Fig. S5), finding that ascorbic acid (vitamin C) was a potent inhibitor of thiaminase I activity. The IC_50_ was 266 nM, with complete inhibition of thiaminase activity at concentrations greater than 4.5 µM (Fig. 7a). By HPLC, in the presence of pyridoxine, thiaminase, and thiamine, the expected peak at 3.45 min corresponding to the pyrimidine-pyridoxine adduct was formed and thiamine (retention time = 3.30 min) was depleted (Fig. 7b). With the addition of ascorbic acid to the above composition, thiamine was retained and no product peak was detected (Fig. 7c). The specificity of ascorbic acid inhibition was confirmed by depleting ascorbic acid with ascorbate oxidase and observing the return of thiamine degradation (see Supplementary Fig. S6). We then evaluated the inhibitory kinetics of ascorbic acid, confirming that thiamine degradation was inhibited in a time-dependent and ascorbic acid concentration-dependent manner. The percentage of thiamine degraded decreased with increasing ascorbic acid concentration, but increased with time at all concentrations tested, indicating that this inhibition was reversible (Fig. 7d). This inhibition—which is effective on the order of a few hours—may be a significant factor in dietary thiamine retention, given the time course of passage of thiamine through the GI tract.

**Figure 8:**
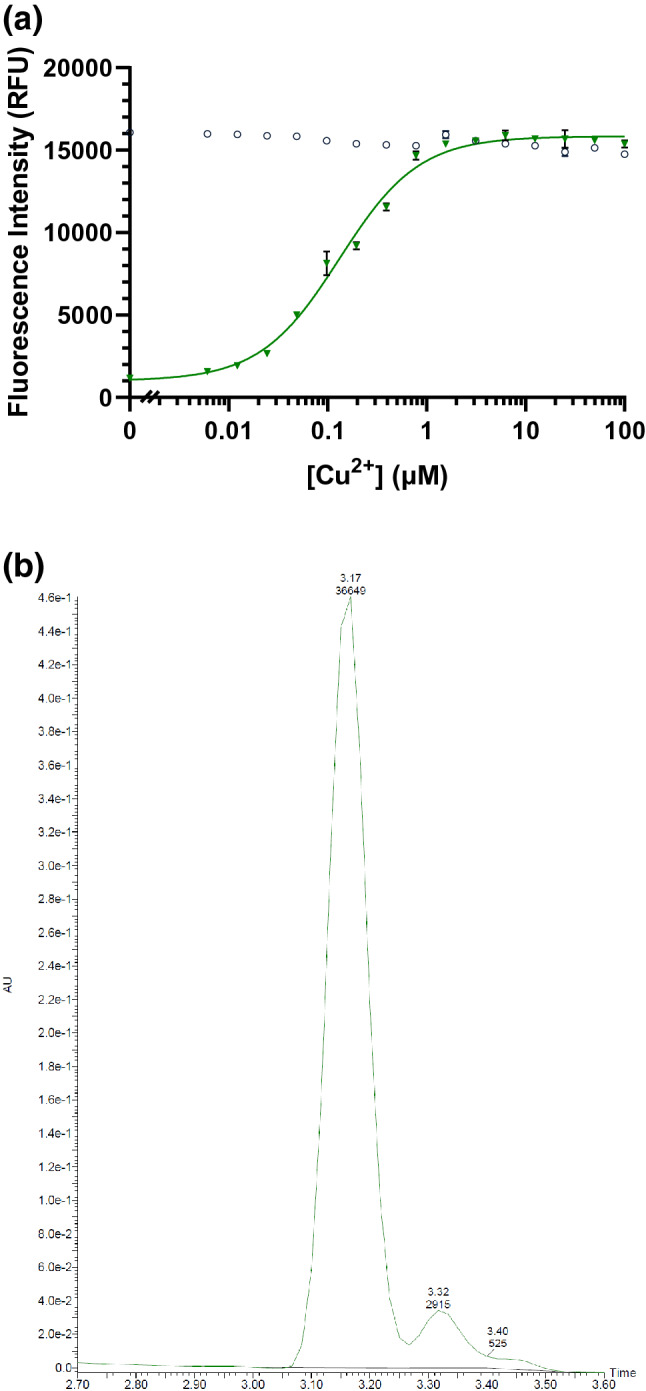

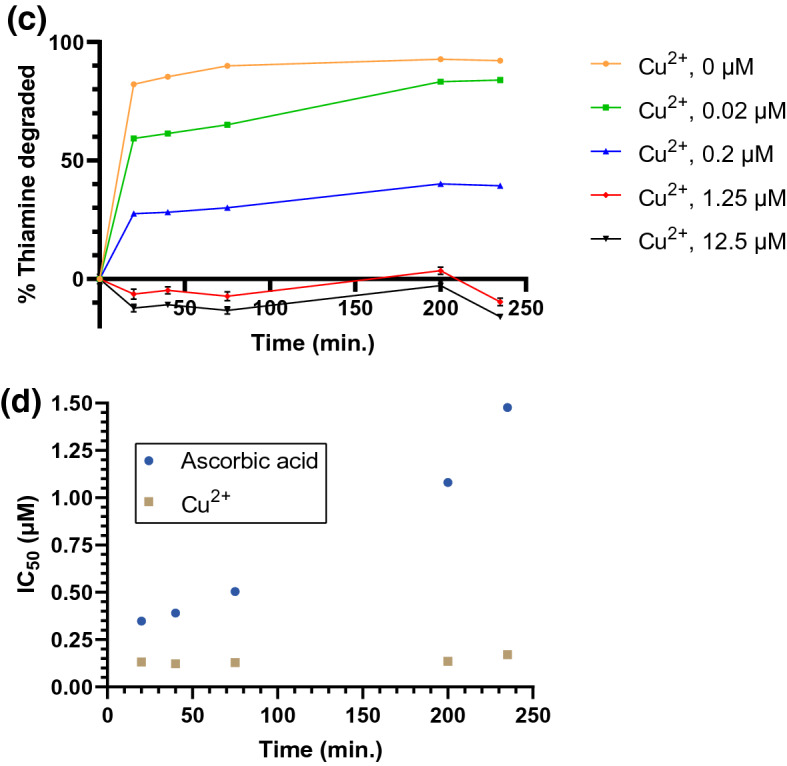
Inhibition of thiaminase I-mediated thiamine degradation in the presence of CuCl_2_ (**a**) Fluorescence intensity measurements made in the presence of 10 µM thiamine, 1 mM pyridoxine, and Cu^2+^ (6 nM–100 µM) in the presence (green triangles) and absence of 0.33 µg/mL thiaminase I (empty black circles) at 37 °C for 1 h. 5 µL of the reaction mixture was diluted with 45 µL water, then 100 µL potassium ferricyanide in 15% (w/v) sodium hydroxide was added prior to measurement using 360/40 nm excitation, 450/50 nm emission. Each point is the average of the fluorescence values in triplicate with min. and max. values represented by the error bars. A 4-parameter logistic equation was used to fit the data (dotted line) from the thiaminase incubations. (**b**) Chromatogram showing the lack of formation of the pyridoxine-pyrimidine adduct at 3.45 min and loss of thiamine (3.30 min) under conditions in (**a**) in the presence of 100 µM Cu^2+^. (**c**) The kinetics of thiamine degradation using endpoint fluorescence intensity measurements at ascorbic acid concentrations ranging from 0 to 12.5 µM from 15 min to 4 h. (**d**) Effect of time on the IC_50_ for ascorbic acid and Cu^2+^.

Cu^2+^ was a more potent inhibitor of thiamine degradation than ascorbic acid, with an IC_50_ of 132 nM for thiaminase activity in the presence of thiamine and pyridoxine and complete inhibition of thiamine digestion at 1 µM (Fig. 8a). Similar to ascorbic acid, thiamine (retention time = 3.30 min) was not depleted with the addition of Cu^2+^ to the above composition of pyridoxine, thiamine, and thiaminase, and the peak corresponding to the pyrimidine-pyridoxine adduct (retention time = 3.45 min) was not formed (Fig. 8b). This indicated that the degradation of thiamine was inhibited. However, in contrast to ascorbic acid, no time-dependent effect of Cu^2+^ on thiaminase inhibition was observed. At a concentration of 1.25 µM or greater, thiamine degradation was fully inhibited and did not recover after 4 h (Fig. 8c). We observed that the IC_50_ remained static for Cu^2+^ through time, though increased over time for ascorbic acid, illustrating the reversibility differences in their inhibitory actions (Fig. 8d).

### Impact of natural constituents on thiaminase I activity from other bacteria

**Figure 9:**
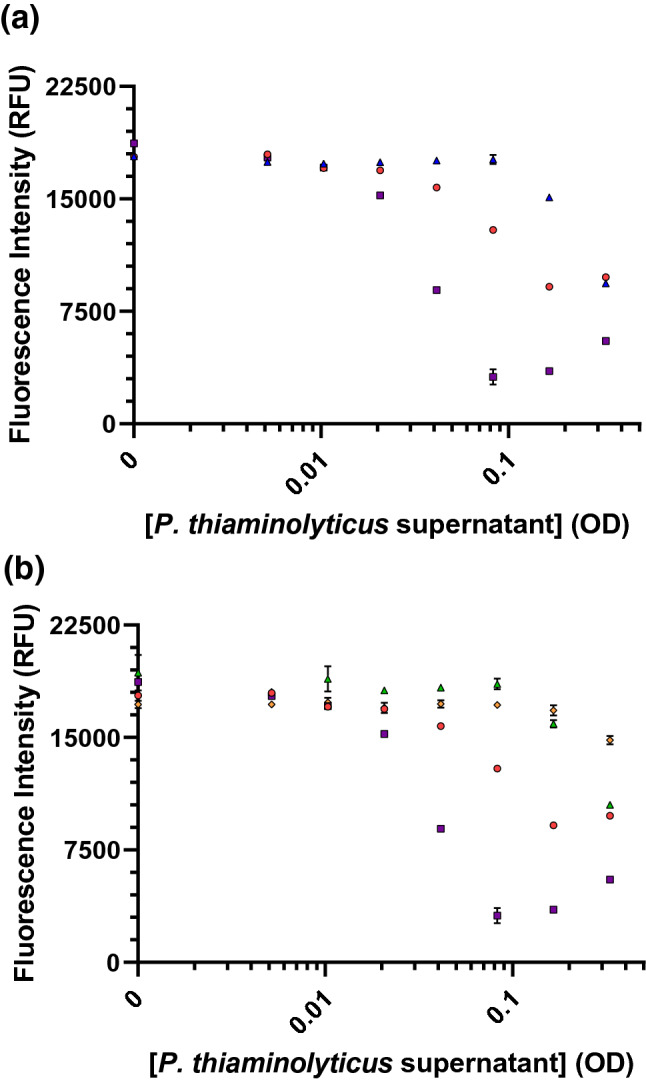
(**a**) Enhancement of *P. thiaminoyticus* thiaminase I-mediated thiamine degradation in the presence of 100 µM pyridoxine (purple squares) and 100 µM nicotinic acid (red circles) in tryptic soy broth versus tryptic soy broth alone (blue triangles). (**b**) Inhibition of *P. thiaminoyticus* thiaminase I-mediated thiamine degradation in the presence of 100 µM CuCl_2_ and 100 µM pyridoxine (green triangles) or 100 µM nicotinic acid (orange diamonds). Fluorescence intensity measurements were made in the presence of 10 µM thiamine, 100 µM pyridoxine or 100 µM nicotinic acid and 0–100 µM Cu^2+^ in the presence of crude *P. thiaminolyticus* supernatants in TSB at 37 °C for 1 h, as described in the “Materials and methods” section. The x-axis corresponds to dilutions of the supernatant from the OD measured of the bacteria at the time of supernatant collection. Each point is the average of the fluorescence values with error bars representing the standard deviation of triplicate measurements.

We extended our investigation to examine thiaminases produced by *B. thailandensis, P. thiaminolyticus, and P. apiarius* and secreted into the culture supernatants as the bacteria grow. Under equivalent conditions for growth, collection and analysis, the thiaminase activity of *P. thiaminolyticus* was approximately eight times greater than *B. thailandensis* and *P. apiarius* using 4-NTP (1.42 nmol thiamine degraded/min, Fig. S7), a known effective co-substrate for diverse thiaminase I enzymes^27,43,54^. The activity of the latter two supernatants were roughly equivalent to each other and to a tenfold dilution of *P. thiaminolyticus* (0.17 ± 0.05 nmol thiamine degraded/min). The complexity of the conditioned culture medium did not permit quantitative and discreet qualitative assessments of enzyme activity or a direct concentration for comparison of the secreted thiaminases. However, for all bacterial supernatants, the thiamine degradation activity was augmented by pyridoxine and inhibited by Cu^2+^. To digest an equivalent amount of thiamine in culture supernatant from *P. thiaminolyticus* diluted in sterile TSB, approximately four and eight times less thiaminase would be needed in the presence of 100 µM nicotinic acid and pyridoxine, respectively (Fig. 9a). For *B. thailandensis*, no degradation of thiamine was observed in culture supernatant diluted in sterile TSB in the absence or presence of nicotinic acid, but the addition of pyridoxine permitted degradation (Fig. S8). Negligible degradation (~ 6%) by *P. apiarius* was observed in the highest concentration of supernatant tested and only with the addition of pyridoxine. It is of note that TSB itself was inhibitory to thiaminase activity with thiaminase added from either *C. botulinum* or *P. thiaminolyticus* sources (Fig. S9).

We observed that the addition of Cu^2+^ reduced the activity of all thiaminases in the crude supernatants (Fig. 9b, Figs. S8, S10), suggesting that this inhibition is conserved across thiaminases. At low concentrations of crude thiaminases from *P. thiaminolyticus* and high concentrations from *P. apiarius* in TSB, we observed a small, but statistically significant inhibition by 100 µM ascorbic acid (Fig. S11). However, we did not observe the inhibition of enzyme activity by ascorbic acid at high concentrations of any crude supernatants or purified *C. botulinum* in this complex culture media. Ascorbic acid can form complexes or become oxidized^56–58^ and this may impact its accessibility or stability in TSB. The presence of complex media also brings numerous potential inhibitors and cofactors, all of which may impact the stoichiometry and kinetics required for thiaminase inhibition. Overall, our results, including this concentration-dependent inhibition, speak to likely complexities of thiaminase-activity in varied diets, with the efficiency of thiamine digestion dependent on the source, contact time, and the concentrations of the enzyme, thiamine, co-factor composition and their respective stoichiometries all playing roles.

## Discussion/conclusions

Early studies on thiamine deficiency in captive foxes were associated with raw fish diets, with the cause of thiamine destruction referred to as the ‘fish factor’ or ‘fish principle’ before the conclusive determination of its enzymatic nature^59,60^. Identification of the thiamine cleavage products and susceptibility to heat indicated an enzyme source, which was then studied extensively in the 1940s and 1950s^60^. This enzyme required a nucleophilic co-substrate to carry out thiamine cleavage. For the *C. botulinum* thiaminase, we found that among the natural organic compounds tested, only pyridoxine and nicotinic acid showed significant activity, with pyridoxine acting as a far more efficient co-substrate. Negligible amounts of thiamine were degraded in the presence of thiaminase and the commercially available Vitamin B1 formulation, contrasting with complete degradation with the addition of pyridoxine. In experiments with the B-Complex formulation, approximately half of the thiamine could be degraded in the formulation, with the other half degraded with additional exogenous co-substrate. This illustrated the need for sufficient concentrations of the co-substrate to be present for thiaminase I to have a significant impact on thiamine levels. Exogenous pyridoxine was also a better co-substrate than nicotinic acid for thiaminase I enzymes released by *B. thailandensis, P. thiaminolyticus, and P. apiarius* cultured in crude tryptic soy broth. Pyridoxine also augmented or was required for thiamine degradation in these complex mixtures. This was of note as *P. thiaminolyticus* cultures grown in complex media have been employed for thiaminase-mediated thiamine depletion of foods in animal feeding studies^44–46^. Without further co-substrate additions or high levels of thiaminase produced by the bacteria under the culture conditions employed, the efficiency of thiamine depletion from foods in such studies may be blunted.

Our results also indicated that having sufficient co-substrate concentrations was not the only chemical factor dictating the efficacy of thiaminase in complex mixtures. In the multivitamin formulation, no degradation of thiamine was observed despite exogenous pyridoxine addition, suggesting the presence of inhibitors to enzyme activity. We identified several constituents that modulated enzyme activity, notably that copper (Cu^2+^) and ascorbic acid were potent inhibitors of thiaminase activity, while manganese (Mn^2+^) and iron (Fe^3+^) accelerated its action. Our results on Cu^2+^ inhibition and Mn^2+^ activation in the *C. botulinum* thiaminase are consistent with results from early studies in thiaminases found in carp viscera, fresh water mussel, and whole homogenized fish^30,60,61^. Neither the divergent effects of Fe^2+^ and Fe^3+^ nor the inhibitory effects of ascorbic acid on thiaminase activity have been previously reported, other than a published personal communication from a hatchery manager noting a significant improvement survival in fry experiencing Early Mortality Syndrome through immersion in a bath of vitamin C^62^.

In our experiments, Cu^2+^ was an irreversible inhibitor of thiaminase I activity from *C. botulinum*. The thiaminase I from *C. botulinum* shares structural similarity with group II periplasmic binding proteins common to transport of small molecules for gram-negative bacteria. However, this protein uniquely has enzymatic activity not retained by other members of this protein family^63,64^. Cysteine residues have been identified as active site nucleophiles in the degradation of thiamine by several thiaminases^55,63,65,66^, and, notably, mutation of a cysteine residue (C143S) in the *C. botulinum* thiaminase and a cysteine residue (C113S) in the *P. thiaminolyiticus* thiaminase abolished their activity^55,63^. It has been postulated that cysteine residues can chelate certain transition metals, noting that copper chloride has been shown to inactivate botulism neurotoxin serotype A^67^. In this neurotoxin, mutation of a cysteine residue (C165) was found to negate the inhibitory actions of copper in this metalloprotease^67^. At the organism level, it could be extrapolated that the presence of dietary copper may confer some resistance to thiaminase I-mediated thiamine degradation.

The finding that ascorbic acid was an efficient inhibitor of thiaminase I was particularly notable as this is an essential nutrient in both humans and salmonine fishes^68,69^, unlike many other organisms that are capable of its synthesis. Thiamine deficiency is common in humans whose diets consist primarily of white rice which is devoid of both thiamine and ascorbic acid^70^. Salmonine fishes, including lake trout, Atlantic salmon, and Baltic salmon, are particularly prone to thiamine deficiency and supplementation of hatchery-reared salmonines with vitamin B1 has been widely employed to avoid mortality^71–75^. Rainbow smelt and anchovies, both of which are prey in diets of salmonine predators with offspring that have experienced thiamine deficiency, contain insignificant amounts of ascorbic acid^70^. Ascorbic acid has beneficial impacts on salmonid health, including optimal growth and development, resistance to nitrites, and resistance to bacterial challenges^69,76–78^. The variable dietary intake of ascorbic acid without de novo synthesis and diets predominated by thiaminase-active prey fish may lead salmonine fishes to be particularly susceptible to thiaminase degradation of thiamine and subsequent thiamine depletion. The inhibition of a purified thiaminase I by ascorbic acid was distinct in defined buffer, but we note that more investigations are needed on the relationship between ascorbic acid and inhibition of thiaminase I in complex media.

More broadly, our results indicate that it is important to consider overall dietary composition when considering the impact of thiaminase as a thiamine-degrading constituent. Dietary thiamine will be degraded in the presence of thiaminase when in the presence of appropriate co-substrates, but other dietary constituents will also inhibit its degradation. The lack or presence thereof, respectively, can yield markedly reduced effects on thiaminase activity despite the presence of the enzyme at high levels. Both commonly applied assays to assess thiaminase activity (the 4-nitrothiophenol assay and radiometric assays) supply high concentrations of co-substrate to the reaction, which is similarly supplemented with high concentrations of thiamine^27,43,54,79^. These conditions provide a measure of the maximal thiaminase activity possible in the sample, but are not reflective of the degradation characteristics in the native sample matrix which may lack sufficient levels or appropriate co-substrates or contain inhibitors. We speculate that the diets including significant levels of copper or l-ascorbyl-phosphate as a source of ascorbic acid^44–46^, which have been employed in feed preparation studies for understanding thiamine depletion effects, may reduce the efficiency of thiamine depletion, confounding laboratory reproductions of the effects and kinetics of thiamine deficiency. At the same time, it is important to be mindful that thiaminase I-mediated thiamine depletion would also deplete nucleophilic co-substrates^80^. As both pyridoxine and nicotinic acid deficiencies are associated with similar neurologic and developmental symptoms attributed to thiamine deficiency^74,81^, including erratic and spiral swimming, hyperexcitability, encephalopathy, and reduced growth^82–84^, depletion of these vitamins should not be overlooked in the overall clinical picture when thiaminase I is implicated.

We note that our experiments were restricted to constituents with significant water-solubility, therefore other constituents present in natural diets may contribute to thiaminase-mediated thiamine degradation. Our observation of thiaminase I inhibition by several ions and another vitamin (ascorbic acid) found in GI tracts and external environments suggests that similar inhibitions might be observed by other common dietary constituents. Additionally, our results primarily focused on the thiaminase I enzyme from *C. botulinum*. Although we observed a similar enhancement of thiaminase I activity by pyridoxine and inhibition by Cu^2+^ with crude supernatants from *B. thailandensis, P. thiaminolyticus, and P. apiarius*, it is known that thiaminases from different sources have different co-substrate specificities^85^ therefore might respond differently to potential inhibitors. Expanding this work on both co-substrates and inhibitors to purified thiaminases from other bacterial, plant, insect, or fish sources would elucidate the universality of these findings.

Thiaminase I is a secreted enzyme that has been postulated to reduce thiamine availability to microbial competitors, predisposing them to harm from other toxins and providing a competitive advantage favoring microbes that can synthesize their own thiamine from precursors^22,86^. However, examples of two microbial enzymes described as having thiamine-degrading properties have been found to function to salvage degraded thiamine and recycle components suited for the complicated synthesis of thiamine^20,23,87^. In fact, some microorganisms preferentially use precursors for thiamine synthesis, and are incapable of uptake of exogenous intact thiamine^23,87^. Thiamine is primarily used to supplement human and animal nutrition and is excreted in the urine in its parent form, though metabolism leads to excretion of pyrimidine and thiazole degradation products^88^. Thiamine is also readily degraded in aquatic environments to components that can be recycled by bacteria for thiamine synthesis^20^. The degradation of thiamine increases the availability of its two primary constituents or their analogues for bacteria, plants, and fungi with enzymes that can salvage these compounds for use in synthesizing thiamine, potentially providing an advantage over organisms that must synthesize thiamine from more fundamental components^20,23^. Recognition of the thiamine salvage and thiamine synthesis functions^20,23,87^ leads to questioning the functional purpose of microbial enzymes reported as thiaminases.

Aside from thiaminase I enzymes utilizing thiamine degradation products for thiamine synthesis, we postulate that the adduct formed from the thiamine pyrimidine ring and natural nucleophilic cosubstrates, such as pyridoxine or nicotinic acid, may favor certain bacteria, either as a preferential substrate or as an inhibitory compound to competitors. It is, for example, known that some degradation products of thiamine are toxic analogues that inhibit enzymatic reactions for which thiamine is a required cofactor^78–82^.

We suggest that the dietary presence of vitamin C and Cu^2+^ may influence the manifestation of thiamine deficiency in animals with diets including thiaminase I. Our investigations do not consider all possible dietary sources of thiaminase I-mediated enhancement or inhibition, nor thermal processing methods used to inactivate thiaminases in pre-prepared foods^89,90^,﻿ but maintain that focusing solely on enzyme activation and inhibition leads to a different perspective of thiaminase I than has been commonly considered in investigations of fish or other animal mortality associated with this enzyme. Although the results reported here largely focus on one bacterial thiaminase I enzyme with thiamine-degrading properties, they provide a new perspective regarding a group of enzymes with widely recognized associations with plants (e.g. bracken fern) and animals (fish, ruminant mammals) that have perplexed researchers since their discovery more than 80 years ago.

## Supplementary Information

Supplementary Information.

## Data Availability

The datasets supporting the conclusions of this article are available upon reasonable request from the corresponding author.
