# Supplementary material for: Dietary factors potentially impacting thiaminase I-mediated thiamine deficiency
Source: Sci Rep. 2023 Apr 28;13:7008. doi: 10.1038/s41598-023-34063-5 (PMC10147936; doi:10.1038/s41598-023-34063-5)
Supplement: Supplementary file 1 — Supplementary Information. [file 41598_2023_34063_MOESM1_ESM.docx]

**Supporting information: Dietary Factors Potentially Impacting Thiaminase I-mediated Thiamine Deficiency**

Katie A. Edwards*^,1,2^, Eileen A. Randall^ϯ,3^, Patricia C. Wolfe^ϯ,1^, Esther R. Angert^2^, Clifford E. Kraft^3^

^1^Department of Pharmaceutical Sciences, Binghamton University, Binghamton, NY, 13902

^2^Department of Microbiology, Cornell University, Ithaca, NY, 14853

^3^Department of Natural Resources and the Environment, Cornell University, Ithaca, NY, 14853

*Expression and purification of thiaminase I*

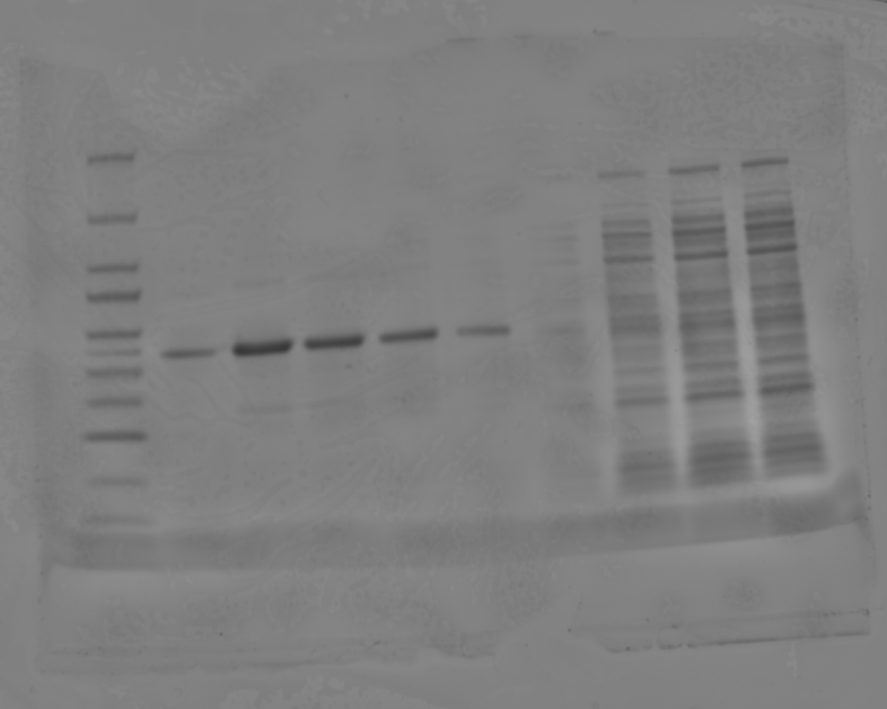
Fig. S1. SDS-PAGE of His-tagged thiaminase I isolated by Ni-NTA-agarose from E. coli total cell lysate. 300 ng - 3 µg of purified thiaminase and 3 – 30 µg of cell lysate were loaded on a 4-15% precast Mini-Protean^®^ TGX polyacrylamide gel. Visualization was afforded using Coomassie staining. The cropped, labeled and enhanced image is shown in the top figure, the complete original gel image is shown in the bottom figure.

*Impact of amino acids, inorganic ions, and vitamins on thiochrome fluorescence*


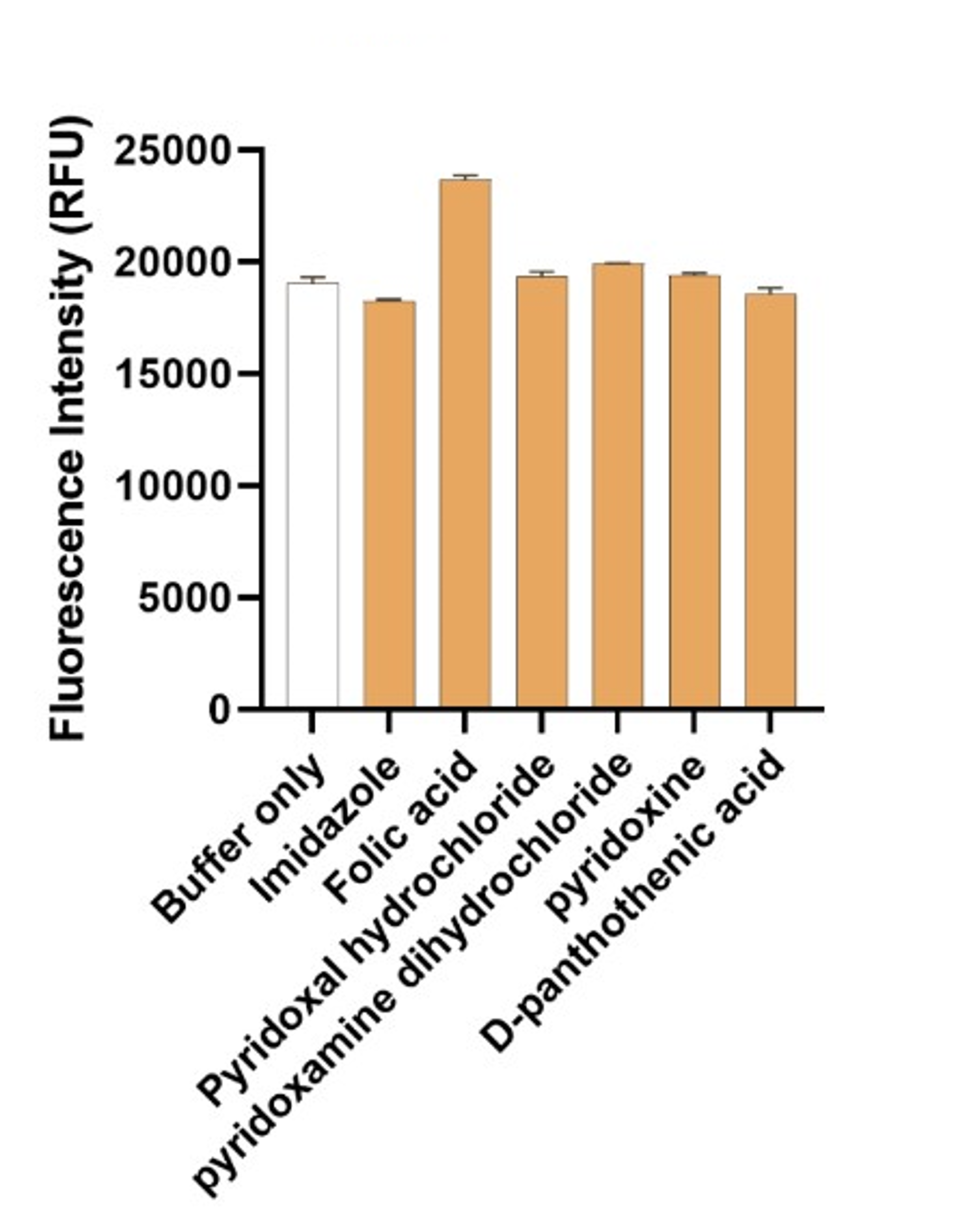

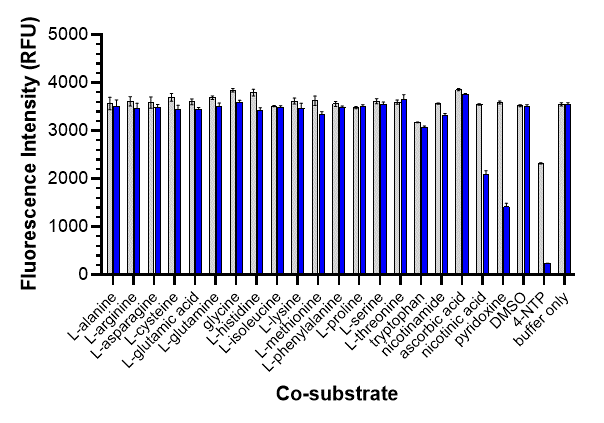
In the absence of thiaminase, there was not a marked impact on thiochrome fluorescence for most natural compounds tested at a 1 mM concentration (Fig. S2). The fluorescence intensity increased (24.6, 8.7 and 8.3%, respectively) for folic acid, ascorbic acid, and glycine versus in buffer only, suggesting an increase in thiochrome conversion efficiency, fluorescence enhancement, or the prevention of natural thiamine degradation by the former as an antioxidant (Fig. 2 and S2). The fluorescence intensity of thiochrome was lower (-10.5%) with L-tryptophan than in buffer only, potentially suggesting a slight interference with thiochrome formation. The co-substrate efficacy results were interpreted as a percent change of fluorescence (Fig. 2), thus, these raw fluorescence values (Fig. S2) served only as controls in the presence versus absence of thiaminase for the respective co-substrate.


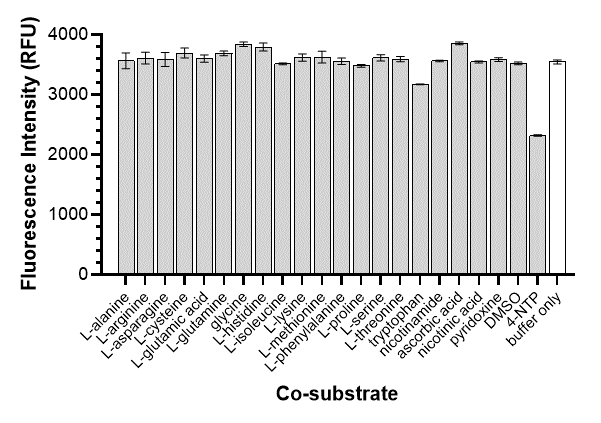
**a.) b.)**

**c.)**

Fig. S2. Fluorescence readings at 360/450 nm following thiochrome conversion in the presence of 10 µM thiamine and 10 mM amino acids or vitamins in 20 mM MES/20 mM NaCl, pH 6.5 a.) and b.) without thiaminase and c.) both with and without thiaminase.

*Multivitamin Composition*

Table S1. Composition of multivitamin tablets

|  | **Central-Vite**^1^ | **Balanced B-100**^2,3^ |
| --- | --- | --- |
| Thiamine (Thiamine Mononitrate) | 1.5 mg | 100 mg |
| Riboflavin | 1.7 mg | 100 mg |
| Niacin (Niacinamide) | 20 mg | 100 mg |
| Pyridoxine Hydrochloride | 2mg | 100 mg |
| Folic Acid | 400 µg | 400 µg |
| Cyanocobalamin | 6 µg | 100 µg |
| Biotin | 30 µg | 100 µg |
| Pantothenic Acid (Calcium Pantothenate) | 10 mg | 100 mg |
| Choline Bitartrate | — | 100 mg |
| Inositol | — | 100 µg |
| Para-Amino Benzoic Acid | — | 30 mg |
| Vitamin A (71% Vitamin A Acetate, 29% beta-Carotene | 3500 IU | — |
| Vitamin C (Ascorbic Acid) | 60 mg | — |
| Vitamin D-3 (Cholecalciferol) | 1000 IU | — |
| Vitamin E (di-alpha Tocopheryl Acetate) | 30 IU | — |
| Vitamin K (Phytonadione) | 25 µg | — |
| Calcium (Calcium Carbonate and Dicalcium Phosphate | 200 mg | — |
| Iron (Ferrous Fumarate) | 18 mg | — |
| Phosphorus (Dicalcium Phosphate) | 20 mg | — |
| Iodine (Potassium Iodide) | 150 µg | — |
| Magnesium (Magnesium Oxide) | 50 mg | — |
| Zinc (Zinc Oxide) | 11 mg | — |
| Selenium (Sodium Selenate) | 55 µg | — |
| Copper (Cupric Sulfate) | 0.5 mg | — |
| Manganese (Manganase Sulfate) | 2.3 mg | — |
| Chromium (Chromium Picolinate) | 35 µg | — |
| Molybdenum (Sodium Molybdate) | 45 µg | — |
| Chloride (Potassium Chloride) | 72 mg | — |
| Potassium (Potassium Chloride) | 80 mg | — |
| Silicon (Calcium Silicate) | 2 mg | — |
| Tin (Stannous Chloride) | 10 µg | — |
| Vanadium (Sodium metavanadate) | 10 µg | — |
| Nickel (Nickel Sulfate) | 5 µg | — |

^1^ Rite-Aid Central-Vite Multivitamin/Multimineral Supplement with Antioxidants. Other ingredients: Microcrystalline cellulose, gelatin, croscarmellose sodium, stearic acid, polyvinyl alcohol, titanium dioxide, polyethylene glycol, magnesium stearate, silicon dioxide, FD&C yellow #6 lake.

^2^ Rite-Aid Natural Balanced B-100 B Complex Formula High Potency. Other ingredients: Cellulose.

^3^ Also tested a thiamine supplement: NOW B-1 100mg Nervous System Health, which was composed of 100mg thiamine. Other ingredients: Cellulose, stearic acid, magnesium stearate.

*Fluorescence Emission Spectra of Multivitamins*


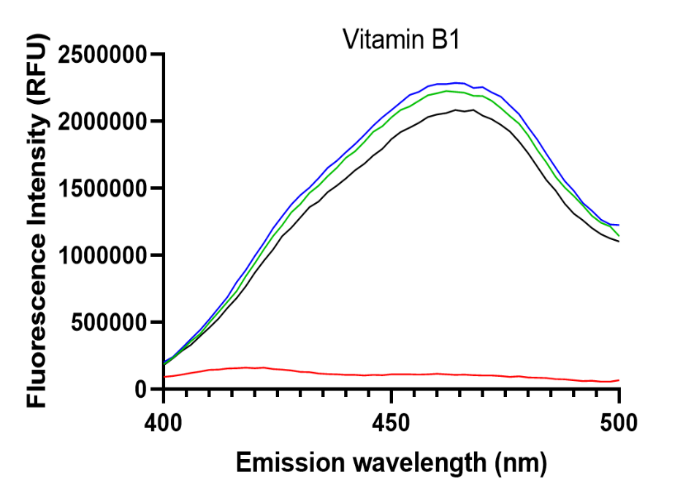

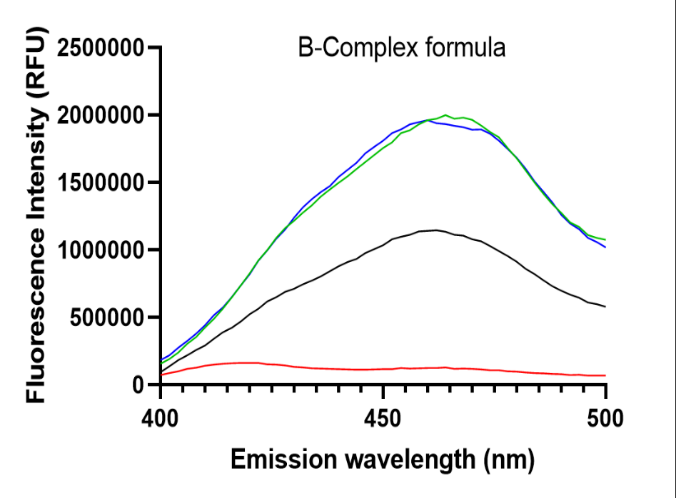


**a.) b.)**


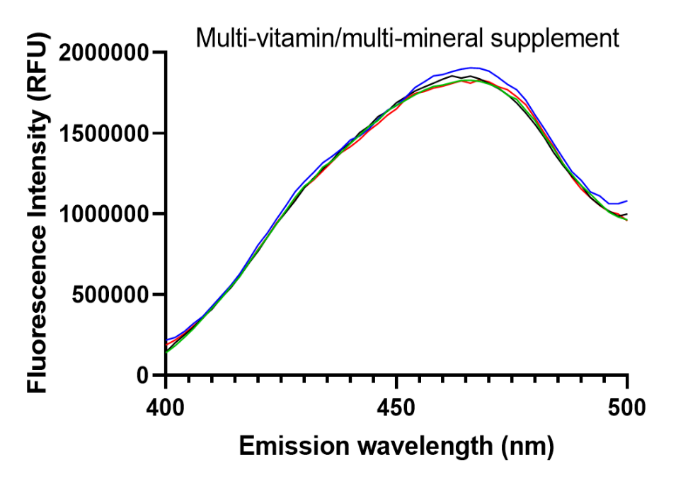


**c.)**

Fig. S3. Fluorescence emission spectra of commercial vitamin B1, B-complex, and multivitamin formulations extracted using simulated gastric fluid, then diluted to theoretical 1 µM thiamine and incubated with (green) 0 µg/mL thiaminase and 0 µM pyridoxine, (blue) 0 µg/mL thiaminase and 100 µM pyridoxine, (black) 0.33 µg/mL thiaminase and 0 µM pyridoxine presence and (red) 0.33 µg/mL thiaminase I and 100 µM pyridoxine at 37ºC for 2 hours prior to oxidation with alkaline ferricyanide and fluorescence measurement using 360/40 nm excitation and emission from 400-500 nm

*Chromatographic data*

The retention times and peak areas of the reaction products of thiamine with nicotinic acid and pyridoxine are tabulated below.

Table S2. Retention times of vitamins and thiaminase reaction products

| **Analyte** | **Column Retention Time (min)** | **Peak Height** | **Peak Area** |
| --- | --- | --- | --- |
| **Pyridoxine as Cofactor,**  **- Thiaminase** |  |  |  |
| Thiamine | 3.30 | 29816 | 2323 |
| Pyridoxine | 3.13 | 48869 | 3629 |
| pyridoxine-pyrimidine analog | - | - | - |
| **Pyridoxine as Cofactor,**  **+ Thiaminase** |  |  |  |
| Thiamine | 3.32 | 13761 | 965 |
| Pyridoxine | 3.13 | 27458 | 2071 |
| pyridoxine-pyrimidine analog | 3.47 | 36653 | 2737 |
| **Nicotinic Acid as Cofactor,**  **- Thiaminase** |  |  |  |
| Thiamine | 3.30 | 25471 | 2891 |
| nicotinic acid | 2.48 | 32352 | 2118 |
| nicotinic acid-pyrimidine analog | - | - | - |
| **Nicotinic Acid as Cofactor,**  **+ Thiaminase** |  |  |  |
| Thiamine | 3.30 | 23234 | 1908 |
| nicotinic acid | 2.50 | 24071 | 1954 |
| nicotinic acid-pyrimidine analog | 2.90 | 4785 | 352 |

*Impact of inorganic ions on thiaminase activity*


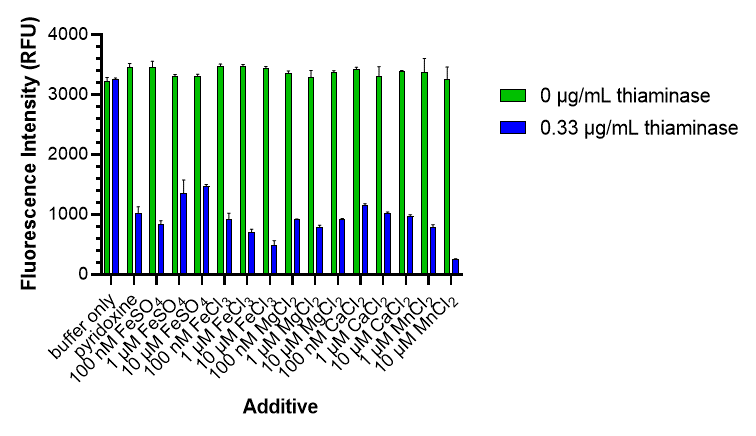
The raw fluorescence values used for calculations referred to in Fig. 6 are shown below as a function of inorganic ion and concentration in Fig. S4.

**a.)**


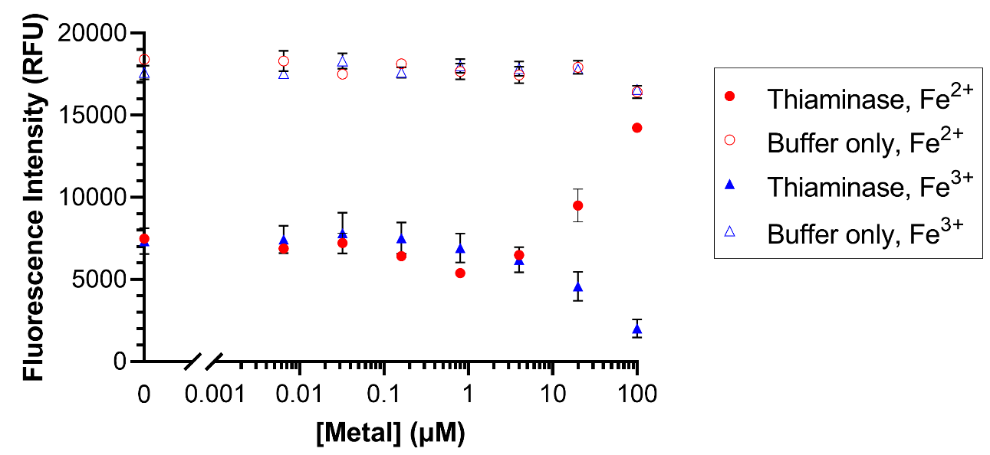
**b.)**


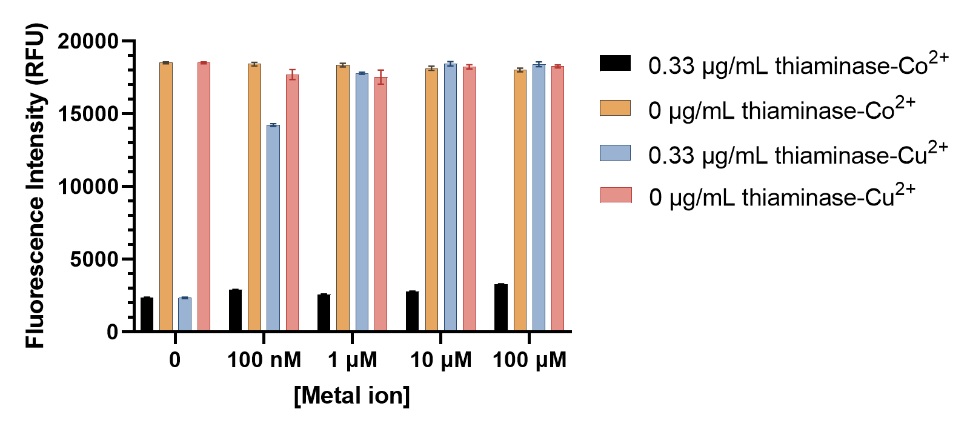


**c.)**

Fig. S4. Effect of inorganic ions on thiamine-depletion by thiaminase I from C. botulinum. 10 µM thiamine, 1 mM pyridoxine and listed inorganic compounds from a.) 0.1-10 µM Fe^2+^, Fe^3+^, Mg^2+^, Ca^2+^, and Mn^2+^ b.) 0.01-100 µM Fe^2+^ (circles) and Fe^3+^ (triangles) and c.) 0.1-100 µM CuCl_2_ and CoCl_2_ in 20 mM MES/20 mM NaCl, pH 6.5 were incubated in the presence and absence of 0.33 µg/mL thiaminase I (solid and open symbols in b., respectively) at 37ºC for 1 hour. 5 µL of the reaction mixture was diluted with 45 µL water, then 100 µL alkaline potassium ferricyanide was added prior to measurement using 360/40 nm excitation, 450/50 nm emission.

*Impact of organic constituents on thiaminase activity*

We screened various amino acids and vitamins for their ability to inhibit thiaminase I when in the presence of both pyridoxine as an optimal co-substrate and thiamine. Ascorbic acid was a potent inhibitor of enzyme activity, with slight inhibition from nicotinamide (Fig. S5).

Fig. S5. Effect of organic constituents on thiamine-depletion by thiaminase I from *C. botulinum.* 10 µM thiamine, 1 mM pyridoxine and listed organic compounds from 0.1-10 µM in 20 mM MES/20 mM NaCl, pH 6.5 were incubated in the presence and absence of 0.33 µg/mL thiaminase I at 37ºC for 1 hour. 5 µL of the reaction mixture was diluted with 45 µL water, then 100 µL alkaline potassium ferricyanide was added prior to measurement using 360/40 nm excitation, 450/50 nm emission. Each bar is the average of the fluorescence values in triplicate with standard deviation represented by the error bars.

*Impact of ascorbate oxidase on restoration of thiaminase activity*

Thiaminase I-induced thiamine degradation was inhibited by ascorbic acid (Fig. S5). We confirmed that this inhibition could be reversed in the presence of ascorbate oxidase (Fig. S6). Ascorbate oxidase converts ascorbic acid (reduced form) to dehydroascorbic acid (oxidized form). The latter would be present as a product of this secondary enzyme reaction, yet, did not inhibit thiaminase 1, indicating that only the reduced form of ascorbic acid served as an inhibitor.

Fig. S6. Return of ascorbic acid-inhibited thiamine-depletion by thiaminase I in the presence of ascorbate oxidase. 1 µM thiamine, 100 µM pyridoxine and 500 µM ascorbic acid were incubated in the presence of 0-5 U/mL of ascorbate oxidase in 20 mM MES/20 mM NaCl, pH 6.5 in the presence and absence of 0.33 µg/mL thiaminase I at 37ºC for 1 hour. 5 µL of the reaction mixture was diluted with 45 µL water, then 100 µL alkaline potassium ferricyanide was added prior to measurement using 360/40 nm excitation, 450/50 nm emission. Each bar is the average of the fluorescence values with the standard deviation represented by the error bars.

*Relative thiaminase activity of supernatants from Burkholderia thailandensis, Paenibacillus thiaminolyticus, and Paenibacillus apiarus*

The supernatants from *B. thailandensis, P. thiaminolyticus,* and *P. apiarus* grown in tryptic soy broth (TSB) were collected following an equivalent time period for growth and sterile filtered. The crude supernatants were assayed for thiaminase activity using the 4-NTP assay.^28^

Fig. S7. Thiaminase activity (nmol/min.) in culture supernatants assayed without dilution using the 4-nitrothiophenol (4-NTP) assay, carried out as previously reported.^27^ The results are an average of three replicates with error bars representing their standard error.


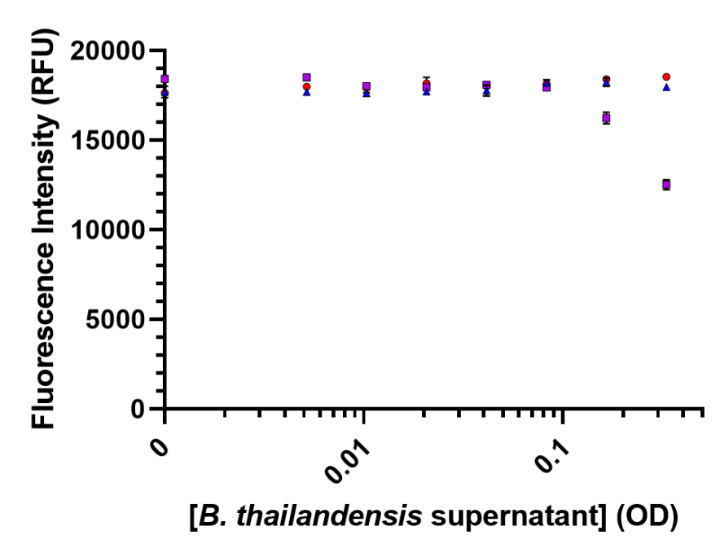
**a.)**

**b.)**

Fig. S8. a.) Enhancement of *B. thailandensis* thiaminase I-mediated thiamine degradation in the presence of 100 µM pyridoxine (purple squares) and 100 µM nicotinic acid (red circles) in TSB versus TSB alone (blue triangles). b.) Inhibition of *B. thailandensis* thiaminase I-mediated thiamine degradation in the presence of 100 µM pyridoxine in TSB in the absence (purple squares) and presence of 100 µM CuCl_2_ (green triangles). The degradation in the absence of CuCl_2_ for pyridoxine was replotted here from Fig. S8a using the same symbols. Fluorescence intensity measurements made in the presence of 10 µM thiamine, 100 µM pyridoxine or 100 µM nicotinic acid, and 0 or 100 µM Cu^2+^ in the presence of crude *B. thailandensis* supernatants in TSB at 37ºC for 1 hour. The x-axis corresponds to dilutions of the supernatant from the OD measured of the bacteria at the time of supernatant collection. 5 µL of the reaction mixture was diluted with 45 µL water, then 100 µL potassium ferricyanide in 15% (w/v) sodium hydroxide was added prior to measurement using 360/40 nm excitation, 450/50 nm emission. Each point is the average of the fluorescence values with error bars representing the standard deviation of triplicate measurements.


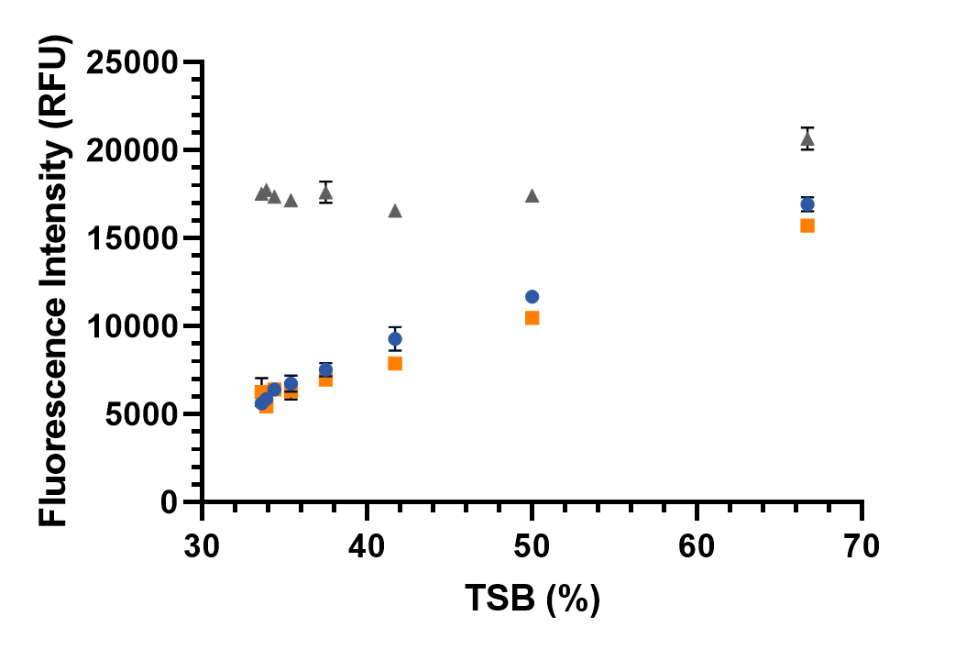


Fig. S9. Thiaminase activity from *C. botulinum* (0.33 µg/mL, orange squares) and *P. thiaminolyticus* (0.167 OD, blue circles) in conditioned culture medium diluted in sterile TSB comprising 33.6% to 66.7% of the overall reaction mixture relative to the same in the absence of thiaminase activity (grey triangles). The remaining percentage of solvent was 20 mM MES, 20 mM sodium chloride, pH 6.5. All mixtures contained 10 µM thiamine and 100 µM pyridoxine and were incubated at 37ºC for 90 minutes. 5 µL of the reaction mixture was diluted with 45 µL water, then 100 µL potassium ferricyanide in 15% (w/v) sodium hydroxide was added prior to measurement using 360/40 nm excitation, 450/50 nm emission. Each point is the average of the fluorescence values with error bars representing the standard deviation of triplicate measurements.

Fig. S10. Inhibition of *P. apiarius* thiaminase I-mediated thiamine degradation in the presence of 10 nM-100 µM CuCl_2_ in the presence (olive squares) and absence of (blue squares) culture supernatant at the equivalent of 2 OD *P. apiarius* with 10 µM thiamine and 100 µM pyridoxine after 1 hour at 37ºC. 5 µL of the reaction mixture was diluted with 45 µL water, then 100 µL potassium ferricyanide in 15% (w/v) sodium hydroxide was added prior to measurement using 360/40 nm excitation, 450/50 nm emission. Each point is the average of the fluorescence values with error bars representing the standard deviation of triplicate measurements.

Fig. S11. Degradation of thiamine by *Burkholderia thailandensis, Paenibacillus thiaminolyticus, and Paenibacillus apiarius* within crude supernatants in TSB in the presence of 10 µM thiamine and 100 µM pyridoxine after one hour at 37ºC. 5 µL of the reaction mixture was diluted with 45 µL water, then 100 µL potassium ferricyanide in 15% (w/v) sodium hydroxide was added prior to measurement using 360/40 nm excitation, 450/50 nm emission. Each point is the average of the fluorescence values in triplicate with min. and max. values represented by the error bars. Significance was assessed using two-way ANOVA with one and three asterisks indicating significance at p=0.05 and p=0.0001, respectively.
